# Intermittent low-dose bevacizumab in hereditary hemorrhagic telangiectasia: A case report

**DOI:** 10.1007/s00508-016-1124-4

**Published:** 2016-11-23

**Authors:** Florian Huemer, Martin Dejaco, Christoph Grabmer, Thomas Melchardt, Daniel Neureiter, Georg Mayer, Alexander Egle, Richard Greil, Lukas Weiss

**Affiliations:** 1 IIIrd Medical Department with Haematology, Medical Oncology, Haemostaseology, Infectious Diseases and Rheumathology, Oncologic Center, Paracelsus Medical University, Muellner Hauptstraße 48, 5020 Salzburg, Austria; 2 Salzburg Cancer Research Institute with Laboratory of Immunological and Molecular Cancer Research and Center for Clinical Cancer and Immunology Trials, Salzburg, Austria; 3 Cancer Cluster Salzburg, Salzburg, Austria; 4 Department of Otorhinolaryngology, Paracelsus Medical University, Salzburg, Austria; 5 Blood Group Serology and Transfusion Medicine, Paracelsus Medical University, Salzburg, Austria; 6 Institute of Pathology, Paracelsus Medical University, Salzburg, Austria

**Keywords:** VEGF, Anemia, Nosebleeds, Telangiectasia, Transfusion, Bevacizumab

## Abstract

**Background:**

Hereditary hemorrhagic telangiectasia is an inherited autosomal dominant disease presenting with recurrent bleeding episodes and iron deficiency anemia due to vascular malformations. Hereditary hemorrhagic telangiectasia is associated with an increased risk of stroke, gastrointestinal bleeding and pulmonary hypertension and life expectancy is significantly reduced. Excess vascular endothelial growth factor (VEGF) plays a key role in the pathophysiology of the disease.

**Case presentation:**

Here we report about a male patient with hereditary hemorrhagic telangiectasia presenting with pulmonary and central nervous system involvement experiencing repetitive nosebleeds, necessitating frequent local cauterization and transfusion of more than 100 units of packed red blood cells. After initiation of temporary therapy with the anti-VEGF antibody bevacizumab at a dosage of 1 mg/kg body weight every 2 weeks, the nose bleeding episodes and the epistaxis severity score significantly decreased and long-lasting transfusion independence was achieved. Reinitiation of low-dose bevacizumab after relapse again proved effective without any documented therapy-related adverse events. In comparison to other reported anti-VEGF antibody protocols in hereditary hemorrhagic telangiectasia, our treatment approach proved to be cost-efficient.

**Conclusion:**

Intermittent low-dose therapy with bevacizumab represents an effective and cost-efficient treatment option for transfusion-dependent patients with hereditary hemorrhagic telangiectasia.

## Introduction

Hereditary hemorrhagic telangiectasia (HHT) is a genetic disorder inherited in an autosomal dominant manner and caused by mutations in the genes encoding endoglin and activin receptor-like kinase type 1 [1, 2]. The syndrome is characterized by the presence of telangiectasia and arteriovenous malformations (AVM) and an increased vulnerability of these vascular structures causing recurrent hemorrhagic episodes. Most patients suffering from HHT initially present with severe recurrent nosebleeds in early childhood requiring local measures, such as electrocoagulation, oral or intravenous iron substitution or even frequent transfusion of packed red blood cells (RBC). The finding of increased plasma levels of vascular endothelial growth factor (VEGF) in HHT patients provided the basis for targeting VEGF in this syndrome of unbalanced angiogenesis as previously reported [3–5].

## Case presentation

In February 2014 a 57-year-old male patient diagnosed with HHT and suffering from recurrent nosebleeds since childhood was referred to our clinic. The definitive diagnosis was established at the age of 43 years by fulfilling each of the 4 proposed Curaçao criteria: (1) spontaneous and recurrent nosebleeds, (2) presence of telangiectasia, (3) presence of AVM and (4) positive family history [6]. In the past years, the nose bleeding episodes had dramatically increased necessitating multiple nasal cauterization and transfusion of more than 100 units of packed RBC between January 2009 and February 2014. At the initial presentation at our clinic, we observed severe anemia with a hemoglobin level of 5.1 g/dl as well as hypoferritinemia (4 µg/l) requiring continuation of RBC transfusions as well as oral iron supplementation. Further endoscopic and imaging measures also revealed disease involvement of the duodenum, lungs and medulla oblongata. Due to the high frequency of nosebleeds associated with symptomatic anemia requiring RBC transfusions every 14 days and multivisceral disease involvement, intravenous systemic therapy with the anti-VEGF antibody bevacizumab was started in March 2014 and administered every 2 weeks at a dose of 1 mg/kg body weight after obtaining the patient’s informed consent including the off-label use of bevacizumab in HHT. Within several days after the first injection of bevacizumab the severity and duration of nosebleeds impressively decreased. The epistaxis severity score (ranging from 0 to 10), a tool to standardize the severity of nosebleeds, was decreased from nine to two and was maintained beyond cessation of anti-VEGF therapy [7]. Bevacizumab therapy had been started at a hemoglobin level of 7.6 g/dl and after the application of 16 cycles (=32 weeks) a hemoglobin level of more than 13 g/dl was achieved. The therapy was stopped in October 2014 whereas oral iron supplementation was continued.

**Fig. 1:**
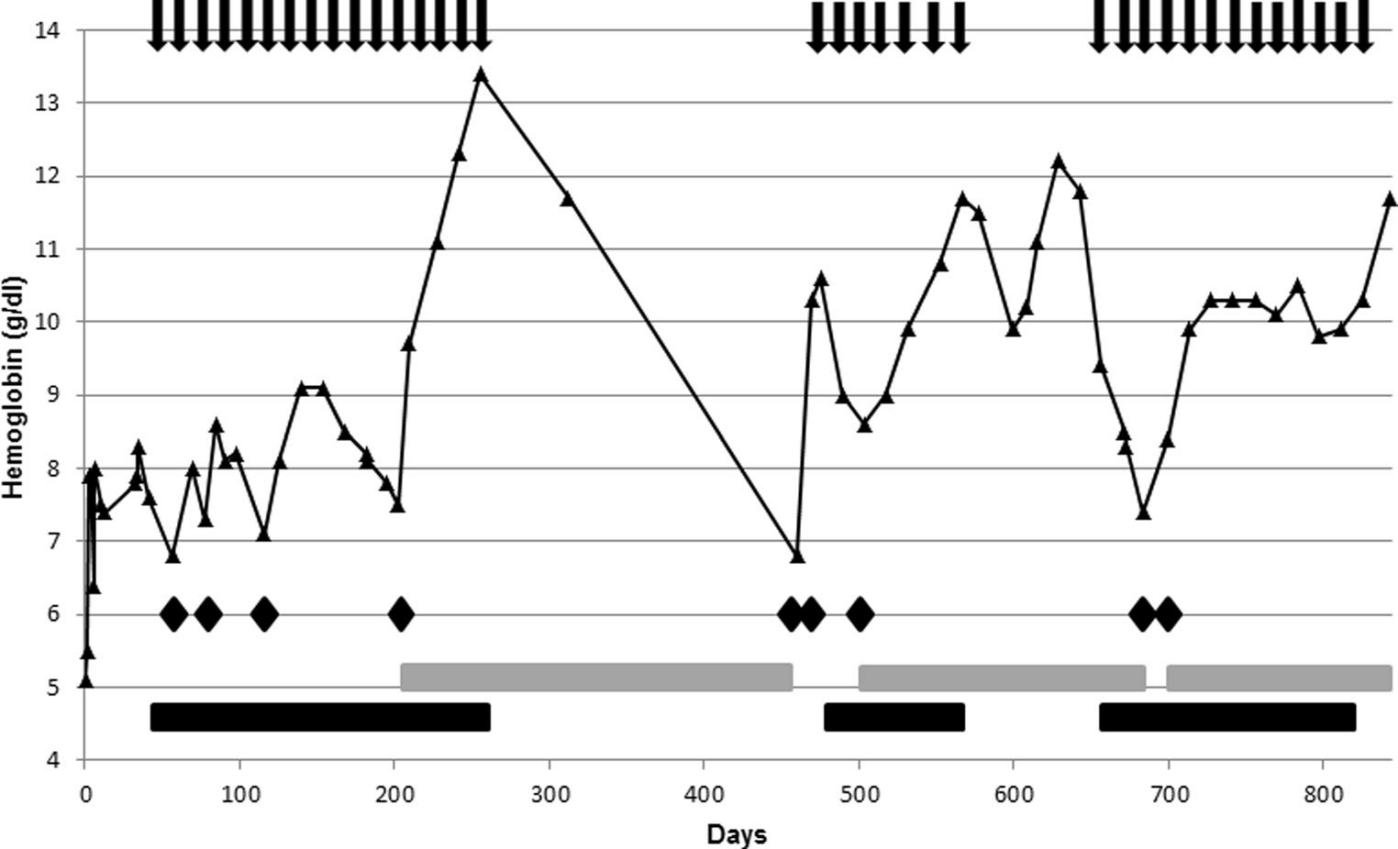
Hemoglobin level during the course of the disease. Initial therapy with bevacizumab started in March 2014 and each *black arrow* indicates a treatment cycle of bevacizumab. The *black bars* illustrate continuous treatment periods with the anti-VEGF antibody whereas the *gray bars* indicate time intervals of transfusion-independence. Each *diamond* represents the transfusion of two packed red blood cells since the start of treatment with bevacizumab

The size of the pulmonary and central nervous system AVM had not changed during anti-VEGF treatment in imaging studies but the nasal mucosa became pale and less vascular. In May 2015, 7 months after stopping bevacizumab, the severity of the nosebleeds gradually increased again and due to symptomatic anemia (hemoglobin 6.8 g/dl) the patient was transfused with RBC again; however, between October 2014 and May 2015 our patient had remained independent of RBC transfusions for 258 days. As a consequence of the recurrence of heavy nosebleeds, we decided to reinitiate therapy with low-dose bevacizumab for another seven cycles in May 2015 which immediately shortened the duration of epistaxis episodes and led to a RBC transfusion independence for a further 181 days. Anti-VEGF treatment was reinitiated in November 2015 due to a progressive relapse of severe nosebleeds. Since January 2016 our patient has been transfusion-independent and anti-VEGF therapy was recently stopped in May 2016 after 13 therapy cycles (Fig. 1). The patient’s epistaxis severity score has currently improved to one. To date, the patient has not experienced any therapy-related adverse events.

## Discussion

Temporary targeting of VEGF with low-dose bevacizumab resulted in an impressive decrease in the severity of epistaxis thereby achieving transfusion-independent intervals of 258 and 181 days on initiation and reinitiation of systemic low-dose bevacizumab therapy and the patient is currently RBC transfusion-independent. Considering the off-label status of bevacizumab in HHT, we decided to use the monoclonal antibody at a lower dose of 1 mg/kg body weight every 14 days in contrast to a dose of 5 or 10 mg/kg body weight as previously reported [4, 5]. Low-dose bevacizumab as treatment for HHT has only been used in a few case reports and case series so far. Thompson et al. prospectively demonstrated an improvement of the epistaxis severity score in a case series with six patients and very low-dose bevacizumab (0.125 mg/kg body weight every 4 weeks) without affecting hemoglobin levels [8]. While several reports show a decrease of the severity and frequency of nosebleeds with low-dose bevacizumab (at doses of 1–2 mg/kg body weight applied every 2–3 weeks), rapid relapses are reported after cessation of anti-VEGF therapy without reporting about long-term follow-up [9, 10]. The effectiveness of continued low-dose bevacizumab treatment (1 mg/kg body weight every 3 weeks) was demonstrated in an HHT patient who had initially been treated with bevacizumab at 5 mg/kg body weight and then relapsed [11].

The total costs of our treatment approach over a time course of 2 years and according to Austrian prices were less than half compared to the costs for bevacizumab therapy with 5 mg/kg body weight (€ 20,263.88 versus € 43,422.60) [5]. The estimated costs of continued RBC transfusions over the same time with a transfusion interval of 14 days would have been € 13,728.00. This treatment approach might pose a threat to patients in terms of the development of alloantibodies and an increased risk of infections. When comparing the costs between a continued low-dose bevacizumab regimen and an intermittent low-dose bevacizumab therapy over 2 years (€ 37,632.92 versus € 20,263.88), the latter proves to be cost-efficient and causes the least cumulative drug exposure [11]. Before initiating treatment with bevacizumab, we assessed the patient history for previous thromboembolic events, diabetes mellitus and age (>65 years) and weighed the potential benefit against a significantly increased risk of thromboembolism. We monitored potential bevacizumab-induced side effects by daily blood pressure measurements and biweekly urinalyses for proteinuria.

As increased plasma levels of VEGF play a key role in the pathophysiology of HHT, several other monoclonal antibodies that target the VEGF signalling pathway, such as aflibercept and ramucirumab or downstream VEGF tyrosine kinase inhibitors, such as cediranib or sunitinib might serve as potential treatment approaches in HHT; however, so far, only one case report demonstrating a reduction of the epistaxis frequency and intensity in an HHT patient during treatment with sunitinib (37.5 mg orally once daily in a 4‑week on/2-week off schedule) for metastatic renal cell carcinoma has been published without reporting on adverse events, such as hand-foot syndrome or gastrointestinal side effects. It is noteworthy that the benefit of epistaxis control decreased during the 2‑week off treatment period [12]. Among VEGF or VEGF receptor targeting monoclonal antibodies, aflibercept has been associated with an increased incidence of hypertension in comparison to bevacizumab and ramucirumab in the treatment of metastatic colorectal cancer [13].

In summary, we show that intermittent low-dose systemic therapy with bevacizumab yields satisfactory transfusion-independent time intervals and demonstrates a valuable treatment option for HHT patients who suffer from severe bleeding episodes. Therapy responses can also be expected on retreatment with low-dose bevacizumab in relapsing patients. Apart from economic considerations, intermittent low-dose bevacizumab therapy might reduce the risk of known dose-dependent anti-VEGF-induced side effects, such as hypertension, proteinuria and bleeding [14].
